# 

**Published:** 1889-02

**Authors:** 


Volume LVIII.] FEBRUARY, 1889. [Number 2.

### The CHICAGO MEDICAL JOURNAL AND EXAMINER

EDITOR:

S. J. JONES, M.D., LL.D.

PUBLISHED MONTHLY UNDER THE AUSPICES OF
THE CHICAGO MEDICAL PRESS ASSOCIATION.

Entered at the Post Office at Chicago, for transmission through the mails as second-class matter.

